# Can the non-identity theory answer the original criticisms of causal theories of spacetime?

**DOI:** 10.1007/s13194-026-00728-x

**Published:** 2026-03-13

**Authors:** Rasmus Jaksland, Niels Linnemann

**Affiliations:** 1 Department of Philosophy, Princeton University, Princeton, New Jersey USA; 2 Department of Philosophy, University of Geneva, Geneva, Switzerland

**Keywords:** Causal set theory, Causal theory of time, Causal theory of spacetime, Causality, Identity theory

## Abstract

Recently, Baron and Le Bihan (2023) have suggested revisiting the causal theory of spacetime, noting that advancements in physics, particularly causal set theory, and metaphysics offer new counters to the criticisms of Smart (1969) and Earman (1972)—criticisms that, they say, defeated the original causal theory of spacetime. Baron and Le Bihan propose that, while the traditional identity causal theory, which seeks to equate spacetime relations with causal relations, is still inadequate, a revised approach could succeed if identity is replaced by grounding and if the grounding base is that of causal set theory. This non-identity causal theory of spacetime posits, in other words, that spatiotemporal relations are grounded in the fundamental causal relations associated with causal sets—in alleged line with the original aim of understanding spacetime through causality. In this paper, we argue that the new arguments put forward by Baron and Le Bihan do little to meet the challenges of Smart and Earman with the result that their non-identity theory does not fare better in addressing these original criticisms than the identity theory. To this end, we will demonstrate that their new arguments make assumptions that (1) are not shared in these historical criticisms of causal theories of spacetime, and, at the same time, (2) are either indecisive or false. Furthermore, we argue that, even with these assumptions, the identity theory can still do just as well as the non-identity theory.

## Introduction

Recently, Baron and Bihan (2023) have suggested revisiting the causal theory of spacetime (Reichenbach, 1956; Van Fraassen, 1970; Grünbaum, 1973), noting that advancements in physics, particularly causal set theory, and metaphysics offer new counters to the criticisms of Smart (1969) and Earman (1972)—criticisms that, they say, defeated the original causal theory of spacetime. Baron and Bihan (2023) propose that, while the traditional *identity* causal theory, which seeks to equate spacetime relations with causal relations, is still inadequate, a revised approach could succeed if identity is replaced by grounding and if the grounding base is that of causal set theory. This *non-identity* causal theory of spacetime posits, in other words, that spatiotemporal relations are *grounded* in the fundamental causal relations associated with causal sets—in alleged line with the original aim of understanding spacetime through causality.

In this paper, we argue that the new arguments put forward by Baron and Le Bihan do little to meet the challenges of Smart and Earman^1^ with the result that their non-identity theory does not fare better in addressing these original criticisms than the identity theory. To this end, we will demonstrate that their new arguments make assumptions that (1) are not shared in these historical criticisms of causal theories of spacetime, and, at the same time, (2) are either indecisive or false. More concretely, on (1), our assessment provides an important qualification to their claim that their project “remains continuous with the historical project of understanding spacetime in causal terms" (Baron & Bihan, 2023, 21). If this programmatic continuity includes those original criticisms of the causal theory of spacetime that they themselves say “was by and large successful" (Baron & Bihan, 2023, 2), then their proposal—their non-identity causal theory of spacetime—does not fulfil the aspiration of such continuity. With respect to (2), we suggest that their change in assumptions offers ways of reviving the identity theory, too, and that their argument that, because of background radiation, “actual signals are systematically propagating between any timelike separated events" (Baron & Bihan, 2023, 5) is not sound.

The paper starts by introducing Baron and Le Bihan’s causal set theory-based non-identity causal theory of spacetime (Section 2). As part of that, we recall two important limitations to this account, namely that it can at most ground spatiotemporal relations in those spacetimes that can be approximated by causal sets; and that the relations in causal sets are causal in the sense of general relativity, i.e., relations of possible causation. Section 3 recalls that Earman’s concern with a causal theory of spacetime was with spacetimes with closed timelike curves and that such spacetimes cannot be approximated by causal sets. Thus, no causal set theory-based causal theory of spacetime can answer Earman’s concern. The next section (section 4) revisits Smart’s objection that he can conceive of an acausal, yet spatiotemporally related event. Baron and Le Bihan reply that such events are non-actual because of background radiation. Where Smart took the modal scope of a causal theory of spacetime to be conceivability, Baron and Le Bihan limit themselves to actuality. The section also emphasises how Smart demanded that the causation of a causal theory of spacetime should be actual causation and not mere possible causation. Baron and Le Bihan seem to accept this demand, but this is in tension with them basing their causal theory of spacetime in the relations of *possible* causation from causal set theory. We take it, though, that Baron and Le Bihan see the pervasiveness of background radiation as an argument for why all the relations of possible causation in causal set theory are relations of actual causation, at least in a causal set of the actual world. Section 5 shows, however, how ungrounded the claim of the pervasiveness of radiation is even for the actual world. Baron and Le Bihan's new non-identity theory can, therefore, not deliver on the demands for modal scope and actual causation in the traditional debate over causal theories of spacetime. Their non-identity theory might still be an improvement on identity theories. In particular, Baron and Le Bihan argue that spacelike but not causally related events are a problem for identity theory. Section 6 argues how identity theories might respond to this problem including being more permissive about what counts as causal relation and that the spacelike relation between two events might not have to be identified with a causal relation between those same events.

In the broader perspective, we conclude that any causal set theory-based causal theory of spacetime will (i) have a hard time meeting Smart’s demand for actual causation and (ii) have a narrower modal scope than what was historically demanded of causal theories of spacetime by, at least, Smart and Earman.

## The non-identity causal theory of spacetime

Baron and Le Bihan recount two versions of the causal theory of spacetime that they, respectively, call “strong identity theory" and “weak identity theory" (Baron & Bihan, 2023, 3). The stronger version defines spatiotemporal relations as causal relations, aiming for a conceptual analysis of spacetime in causal terms. In the weak identity theory, by contrast, spatiotemporal relations are proposed to be identical to causal relations in the sense of the necessary a posteriori: on this view, the concepts ’spatiotemporal relation’ and ’causal relation’ do not have to have the same intension but only the same extension—as long as they have the same extension by necessity. Baron and Le Bihan give the example that spatiotemporal relations and causal relations, on this view, are identical in the way that water is identical to H$_2$O: “Just as the concepts of water and H2O can differ radically, so too for the concepts of spacetime and causation" (Baron & Bihan, 2023, 3-4).

With their non-identity theory, Baron and Le Bihan instead seek to ground—a strictly weaker notion than that of identifying—spatiotemporal relations in causal relations. By ‘grounding’ Baron and Le Bihan quite openly mean any sort of relation that (1) denies identity, and (2) tracks ontological dependence. They explain that they “use the term ‘grounding’ as a general way of referring to a variety of possible relations that may or may not align with the standard use of the term" (Baron & Bihan, 2023, 12). What is important for their purposes is that the grounding relation can obtain in cases where identity does not obtain. Specifically, one can ground relations in absence, they argue, but not identify relations *with* absence—a tenet which becomes relevant for spatially related events that cannot be causally related on the understanding of causation that Baron and Le Bihan adopt.

The other new move in Baron and Le Bihan’s approach is to explicate the causal relation in terms of the models of causal set theory. Causal set theory is, at least programmatically intended as, an approach to quantum gravity.^2^ The models in question, causets, are the kinematic states of causal set theory.^3^ In this approach to quantum gravity, causets are meant to replace Lorentzian spacetimes (in the technical sense of the pair of manifold and Lorentzian metric (*M*, *g*)) at higher energies. Causets, $\mathcal {C}$, are defined as discrete sets of events, *E*, with a partial order, *≺ *, that is reflexive, antisymmetric, transitive, and locally finite. The models can thereby be presented as graphs where the nodes mark events and the links instances of the *≺ * relation.

General relativistic spacetimes—or at least a subsector of them which shall become important in a moment—are proposed to be (approximately) recoverable from causets in an appropriately formulated continuum limit. Just as molecular configurations can approximate a fluid, discrete causets approximate continuum spacetimes. An important *necessary* condition for a causet, $\mathcal {C}$, to approximate a spacetime (*M*, *g*) is that it must embed into the spacetime in such a way that it agrees with the causal order of that spacetime. The causal order determines which spacetime events *could* influence which other spacetime events. In other words, the causal order is about possible causal influences (thus also known as ‘causal connectibility’ in the literature), and not about whether any causal influence was actually exerted between spacetime events. To make the necessary condition precise, consider two spacetime events *x,y ∈ M*. We write that $x \prec _M y$ if and only if the spacetime is such that *x* *could* influence *y* with a probe travelling at most at the speed of light. For the causet, $\mathcal {C}$, with nodes *a,b ∈ E* to approximate the spacetime, (*M*, *g*), there must be a mapping, $\phi : \mathcal {E} \mapsto M$, such that *a ≺ b* if and only if $\phi (a) \prec _M \phi (b)$. That is, events that are (not) connected on the causal set, i.e., events that (do not) stand in *≺ * relation, must be embedded in such a way that they cannot be connected by causal paths on the manifold as well.^4^ This explains why the approach is called *causal* set theory. The structure of the *≺ * relation in the causet mirrors the causal structure of the spacetime it recovers. The basic heuristic of causal set theory is, thus, that causal set theory provides a way to recover the whole spacetime merely from a discrete mirror of its causal structure. This might be the reason why (Baron & Bihan, 2023, 6) refer to *≺ * as “a relation of causal precedence."

This seems all a promising departure point for a causal theory of spacetime. The idea would be that, for a causet that recovers a spacetime, the spatiotemporal relations of that spacetime might be accounted for in terms of these relations of “causal precedence" in the causet. This is exactly what Baron and Le Bihan set out to do by providing concrete constructions for what they call “timelike" and “spacelike" distance.^5^

“Timelike distances between two spacetime points are grounded in the number of causal links between two causal set elements. The [timelike] distance between two spacetime points in the relativistic description thus corresponds to the shortest path through the causal set, between two causal set elements corresponding roughly to the two spacetime points [...]. The distance between any two spacelike separated points is then grounded in the minimum number of relations of causal precedence it takes to arrive at a common causal ancestor within the underlying causal set description" (Baron & Bihan, 2023, 12).

Here “causal links" and “relations of causal precedence" are different ways of referring to the *≺ * relation (Baron & Bihan, 2023, 6). As argued by Jaksland and Linnemann (2024), both of these accounts face some physical-mathematical difficulties. These, however, will not become relevant until Section 6. For now, what is important is that Baron and Le Bihan propose to ground spatiotemporal distances in a specific chain of nodes in the causet that is selected by an optimisation procedure on the number of links in the chains.^6^

In sum, the salient features of Baron and Le Bihan’s proposal are these: A correspondence between spacetimes and causal sets, a grounding of spatiotemporal relations (first and foremost, that of distances) in particular chains of elements related by the *≺ * relation, and an interpretation of these relations as causal relations such that the proposal as a whole can qualify as a non-identity causal theory of spacetime.

## Earman’s objection

The first of these—the correspondence between spacetimes and causal sets—is already the occasion for the first discontinuity with the original debate over the causal theory of spacetime. The work of Earman is mentioned by Baron and Le Bihan as one of those that defeated the original causal theory of spacetime.^7^ Thus, it must be assumed that they intend their revival of a causal theory of spacetime through their non-identity theory to meet this objection. This, at least, seems to be the suggestion when they, as quoted above, claim that their non-identity theory is “continuous with the historical project of understanding spacetime in causal terms."

Earman’s criticism^8^ builds around a specific spatiotemporal relation, spatiotemporal coincidence, and its reductive analysis on a causal theory of spacetime (as used explicitly by Van Fraassen (1970, 184), that is

*X* and *Y* are spatiotemporally coincident just in case: for every *Z*, *Z* is causally connectible with *X* if and only if *Z* is causally connectible with *Y*. Earman (1972, 77)

As Earman makes clear, this analysis will, among other cases,^9^ fail if there are closed causal curves: if there is a causal loop with *x* and *y* on that loop and non-coincident by assumption, any *z* connectible to *x* will also (via the loop) be causally connectible to *y*—and vice versa—in violation of the spacetime coincidence condition. Such causal loops, however, occur in what seems to be relevant models of general relativity, such as that of a rotating black hole, Kerr spacetime. So, spatiotemporal coincidence cannot be causally analysed in the proposed terms in all GR spacetimes, including rather relevant ones.

Earman does not disprove that another, more subtle causal analysis with, in particular, a different analysis of spatiotemporal coincidence above could still be possible. He suspects, though, that such a causal analysis will in fact be impossible. If this is indeed so, a causal theory of spacetime thus fails to get—in Earman’s words—“at the subtle and complex spatiotemporal relations which can obtain between events set in a relativistic space-time background" (Earman, 1972, 79). Earman’s point reminds us thus that there are complex spacetime models of general relativity where the spatiotemporal relations seem impossible to account for in terms of causal relations (in the sense of general relativity).

Earman’s basic objection is, in other words, that the causal theory of spacetime can at most account for the spatiotemporal relations in a subset of possible GR spacetimes. Earman asks rhetorically: “But precisely what reasons, other than the question-begging one that the causal analysis fails, are there for thinking that such a space-time [not included in that subset] is physically unreasonable?" (Earman, 1972, 78).

What new reply might a non-identity causal theory of spacetime based on causal set theory offer to Earman’s objection? First, let us notice that this concrete spatiotemporal relation, spatiotemporal coincidence, is not one that Baron and Le Bihan propose any ground for, and they do not argue either that no such ground is necessary. Thus, already in this sense, they do not answer the criticism by Earman that they themselves cite as defeating the identity causal theory of spacetime. The more interesting question is, though, whether their non-identity theory could answer Earman’s objection. The problem, Earman explains, arises in spacetimes with closed timelike curves. Thus, any solution to this problem that is based on relations in causal set theory, as would have to be the case for Baron and Le Bihan’s non-identity theory, *requires* that causal set theory can account for spacetimes with closed timelike curves. The problem, however, is that the notion of causal precedence in causal set theory is, qua partial order, simply inconsistent with the possibilities of causal loops^10^: The possibility of a loop means that there is $x \prec y \prec x$ for * x ≠ y*, in clash with the antisymmetry requirement on a causal set (*x ≺ y* and *y ≺ x* implies *x = y*). But it simply cannot: causal set theory is, independent of its interpretation, simply inconsistent with loops.    

An alternative counter to Earman’s criticism has, however, already been proposed in the literature by Wüthrich and Huggett (2025):

The causal theorist is bound to fail if their pretension is to produce an alternative foundation for full GR, as Earman conclusively establishes; however, if the aspiration is instead to offer either a merely ‘localist’ reconstruction of spacetimes in a sector of GR or else—and more relevantly for our present purposes—a distinct, and perhaps more fundamental, theory, Earman’s objections can be circumnavigated.

The solution, in other words, is to simply disagree with Earman that spacetimes with closed timelike curves are spacetimes that a causal theory of spacetime should accommodate. This is a legitimate point of view, but, for present purposes, it would, in our view, simply constitute explicitly rejecting the traditional aspirations of a causal theory of spacetime. Furthermore, this strategy of dismissing rather than solving Earman’s objection is available, too, to identity theory. This seems to question the dialectic of Baron and Le Bihan. After all, their non-identity theory is stated to be motivated by the defeat that the identity theory suffered from the objections of Earman and others. In other words, if Earman’s objection is a reason to reject identity theories, it is a reason to reject causal set theory-based non-identity theories, too. If Earman’s objection is not a reason to reject non-identity theory, it is, for all Baron and Le Bihan say, not a reason to reject identity theory, either.

This marks a recurrent theme below: The types of spacetimes and their relations that Baron and Le Bihan might be able to ground in causal relations with their non-identity theory of spacetime based on causal set theory are limited. The possible modal scope of Baron and Le Bihan’s causal theory of spacetime is much more limited than the modal scope that the original criticism assumed was necessary for a causal theory of spacetime. This does not, of course, defeat the non-identity theory as a causal theory of spacetime, but it is an important qualification of their claim that the theory “remains continuous with the historical project of understanding spacetime in causal terms" (Baron & Bihan, 2023, 21).

## Smart’s objection

One can, of course, object that Baron and Le Bihan do not explicitly claim that their non-identity theory can answer Earman’s objection. Baron and Le Bihan (2023, 3–4) do, however, explicitly discuss Smart’s objections to such a theory.

Among these objections, the central one is this:

It is difficult to see how the causal theory of time is applicable to theories which allow for the existence of events which are neither causes nor effects of other events. It at least seems to me that I can consistently envisage a universe of purely random events spread out through space-time (Smart, 1969, 394, quoted in Baron & Bihan, 2023, 4).

According to Smart, that one can “consistently envisage" events in spacetime that are neither the cause nor the effect of any other event implies that identity theory cannot be correct. In Smart’s presentation, the causal theory of spacetime is defeated because it is possible—in the sense of being conceivable—to have a spacetime with events that, by virtue of being in spacetime, are spatiotemporally related to other events, but that are not causally related to other events. For such events, there are no causal relations that their spatiotemporal relations can be identified with, and the identity causal theory of spacetime therefore fails.

Baron and Le Bihan recount Smart’s objection in this way:

According to the weak identity theory, spatiotemporal relations and causal relations are identified. Thus, any spatiotemporal relation must be a causal connection, which means that there cannot be any entities that are causally idle so long as they bear spatiotemporal relations to other entities (which they must if they are located in spacetime) (Baron & Bihan, 2023, 4).

Notice that Baron and Le Bihan do not repeat Smart’s emphasis on conceivability or what one can “consistently envisage". Rather, they specify the problem in terms of what “there cannot be". To be true to Smart’s worry, the relevant modality of “cannot be" must, however, be that of conceivability. This will be important to keep in mind below.

Baron and Le Bihan denote their rendering of Smart’s objection “the problem of causal indolence" (2023, 4) and suggest that this really comprises three distinct problems for a causal theory of spacetime: “(i) empty spacetime regions; (ii) timelike connected events that are not causally connected, and (iii) spacelike connected events that are not causally connected" (Baron & Bihan, 2023, 4). The latter two, they imply, immediately follow from their rendering of Smart’s objection. Empty regions of spacetime, in turn, are worrisome because "[w]ithin such regions, there doesn’t seem to be anything to connect via causal relations. Accordingly, there do not seem to be any causal connections to which the relevant spacetime relations might be identified" (Baron & Bihan, 2023, 4).

A good illustration of the problem with empty spacetime regions (subproblem (i)) is empty Minkowski spacetime. In this spacetime, there is no matter content (including no radiation), so there is nothing *actually* influencing anything else. One might think that the clue to Baron and Le Bihan’s answer to such empty spacetime regions is the word “seem". Indeed, in empty Minkowski spacetime (regions) there is no event that is not causally connectible to some other events in the relativistic sense outlined above. This will equally be reflected in causets that approximate such empty Minkowski spacetime: any causet that approximates Minkowski spacetime is *not* one of many nodes without links between them. Rather, there must be many links in such a causet to reproduce discretely how any event in Minkowski spacetime has an infinite number of events in its future and past lightcone (Dowker, 2013, 1654)—displaying what Baron and Le Bihan call causal precedence relations. In this light, one might think that the answer to the problem of ‘empty’ spacetime is that even empty spacetime is full of causal (connectibility) relations.

This is, however, not the reply to (i) that Baron and Le Bihan opt for. In reply to this problem, Baron and Le Bihan instead argue that, due to the pervasiveness of background radiation following the big bang, it is “unclear that the matter fields can have a zero value anywhere in the actual world" (Baron & Bihan, 2023, 5).^11^ Their reply is, in other words, that empty spacetime regions are non-actual. Empty spacetime regions are not a problem for a causal theory of the spacetime of the actual world because of the (supposed) pervasiveness of background radiation (call this *the background radiation argument*). With this reply, however, the problem remains unaddressed for all the possible spacetimes, like the empty Minkowski spacetime above, that do feature empty spacetime regions.

They give a clue about why they opt for this answer to (i) when they repeat this point in their reply to the problem of timelike separated events that are not causally related, that is, to subproblem (ii). Again, because of the pervasiveness of background radiation in our universe “actual signals are systematically propagating between any timelike separated events" (Baron & Bihan, 2023, 5). Thus, “[a]s with empty regions of spacetime, it is unclear that situations in which there are timelike, but not causally, separated events can genuinely arise" (Baron & Bihan, 2023, 5). Again, this can seem like an unnecessarily complicated response, considering that any two timelike separated spacetime events with counterparts in the causet approximating that spacetime will, by construction, be linked causally (in the GR sense), and through the relation of causal precedence (in the CST counterpart). Timelike separated events that are not related by relations of causal precedence are simply impossible in the context of GR and causal set theory proper.

When Baron and Le Bihan do not opt for this more principled reply, it is likely because it does not establish “actual signals"^12^ propagating between all timelike separated events. Recall that the *≺ * relation only receives its causal interpretation from the way it mirrors the causal structure (in the general relativistic sense) of these spacetimes. Therefore, when Baron and Le Bihan refer to the *≺ * relation as “a relation of causal precedence," we must remember that this relation of causal precedence is, at the outset, causal in the sense of general relativity, i.e., potential rather than actual causal influence. Any answer to the causal indolence problem in terms of the *≺ * relation is, at the outset, appealing to relations of merely possible causation. In contrast, those events that are connected by background radiation are actually causally connected, or so Baron and Le Bihan seem to argue.

Baron and Le Bihan might be resorting to a reply involving actual causation because Smart is very clear that the “causal connectibility" of relativity theory is not the right concept of causation for a causal theory of spacetime. One must base its “construction on the notion of connectedness, not that of connectibility" (Smart, 1969, 386). This introduces an important qualification to any causal theory of spacetime based on causal set theory, at least if such a theory wants to be continuous—as Baron and Le Bihan suggest they are—with Smart’s rendering of what such a theory must achieve. The relations of *causal* set theory, more particularly, the *≺ * relation, are not causal in the relevant sense for a causal theory of spacetime. The *≺ * relation that Baron and Le Bihan refer to as “causal precedence" and which serves as the basis for their grounding of timelike and spacelike distance is not a causal relation by Smart’s standards. Standing in this relation is necessary to be causal because actual causation is only possible between nodes that are causally connectible (disregarding possible subtleties due to quantum entanglement). It is, however, not sufficient. To meet Smart’s demands for a causal theory of spacetime, the chains of links in the causet that ground spatiotemporal relations must not only observe the *≺ * relation, but we must also make sure that actual causal influences (what they call ‘signals’) propagate along that chain.^13^ And to repeat, Baron and Le Bihan argue that background radiation ensures this (but see our Section 5 for a counter to that too).

Their problem is that this attempt at meeting Smart’s demand for actual causation removes them from the modal scope of Smart’s objection. Smart objects that he can “consistently envisage" spatiotemporally related acausal events, while Baron and Le Bihan answer that, in worlds like the actual world that feature background radiation, there are no such events. Without an argument that all worlds that can be consistently envisaged are worlds with (suitable) background radiation, background radiation will do little to answer Smart’s objection. There is, however, no trace of an argument to this effect in Baron and Le Bihan’s account. In our reading, therefore, Baron and Le Bihan simply disagree with Smart about the modal scope of a causal theory of spacetime. What Smart seems to want is a causal theory that can account for all conceivable spacetimes, whereas Baron and Le Bihan seem to limit their ambitions to a causal theory of the spacetime of the actual world or, at most, a rather restricted number of possible worlds.

One might wonder, however, how good Smart’s concern about conceivability is, as the link between conceivability and possibility is of course standardly contested. In particular, one might simply care about physical possibility (as suggested by physical theories) as opposed to some vague sense of what people can intuitively conceive. On a charitable reading, then, this accounts also for Baron and Le Bihan’s reconstruction in terms of possibility.^14^ Our point is, however, again that, in limiting the modal scope of the causal theory of spacetime to the actual world or anything short of conceivability, Baron and Le Bihan are not proposing a theory that “remains continuous with the *historical project* of understanding spacetime in causal terms," (p. 21) as at least as Smart thinks of it. In other words, Baron and Le Bihan’s non-identity causal theory of spacetime is not a solution to the problem originally raised by Smart against identity causal theories of spacetime.

Does it, however, at least answer Smart’s objection for worlds where such background radiation is pervasive? Smart’s objection, to repeat, is spacetime “events which are neither causes nor effects of other events" or, as Baron and Le Bihan paraphrase Smart, the objection is that, on the *identity* theory, “there cannot be any entities that are causally idle so long as they bear spatiotemporal relations to other entities (which they must if they are located in spacetime) (p. 4)." Baron and Le Bihan’s own assessment is that the pervasiveness of background radiation is a solution to this problem for *timelike* related events, an argument that Baron and Le Bihan (2023, 5) explicitly say is also available to identity theory. They claim, however, that this argument does not answer Smart’s problem or, rather, their rendering of it, when the spatiotemporal relation is spacelike.

This can initially seem puzzling. The problem, according to Smart and Baron and Le Bihan’s paraphrase of Smart, is causally idle events that are nonetheless spatiotemporally related to other events. One might think, therefore, that the point of bringing up the pervasive background radiation is that no event is actually causally idle. However, if the argument is that causally idle events are impossible in worlds where background radiation is pervasive, then there will be no causally idle but spatiotemporally related events, irrespective of whether the spatiotemporal relation between the events under consideration is spacelike or timelike. Indeed, the attentive reader will notice that this is not how Baron and Le Bihan’s argument was recounted above. Rather, they claim that the pervasiveness of background radiation entails that, for every two timelike related events, there is a causal signal propagating between those two events. The argument must indeed take this form when the problem is rendered, as Baron and Le Bihan do immediately after paraphrasing Smart’s objection, as the problem of “(ii) timelike, but not causally, connected events" (Baron & Bihan, 2023, 4). The causal connection must, in this formulation of the problem, be between the same events that are timelike related.

In contrast, in the quoted remark by Smart and in Baron and Le Bihan’s paraphrase of it, the focus is not on a pair of events and the relations between those two events. Rather, the focus is on a single event in spacetime that is not causally related to any other events but which is spatiotemporally related to other events by virtue of its being an event in spacetime. In contrast, in their formulation of the causal indolence problem, Baron and Le Bihan are more restrictive about what causal relations might qualify to account for spatiotemporal relations. This is the last discrepancy we want to highlight between “the historical project of understanding spacetime in causal terms" and the project actually embarked on by Baron and Le Bihan.

Baron and Le Bihan do not comment on this difference, but there might be a reason that they insist that the causal relation must be between the same events that are spatiotemporally related. Remember that Baron and Le Bihan ground both spacelike and timelike distance in chains of links in the causet, observing the *≺ *-relation. These chains are, at the outset, chains of causal connectibility and not actual causation. According to Smart, however, spatiotemporal relations must be accounted for in terms of relations of actual causation and not merely causal connectibility. Thus, to meet Smart’s demand, Baron and Le Bihan must argue that all causet chains grounding spatiotemporal relations like spacelike and timelike distance are actually causal.

The problem is that if not all timelike separated events are actually causally related, then there will be chains of links in the causet that observe the *≺ * relation but which, nonetheless, are not causal relations in Smart’s sense. Thus, if not all timelike related events are actually causally related, then the causal chains that ground spatiotemporal relations might not encode actual causation. This could be why the problem of timelike related events that are not actually causally related is so important to Baron and Le Bihan.

This does indeed fit with the fact that Baron and Le Bihan, after having rejected timelike but not causally related events, do not comment on whether the *≺ * or “causal precedence" relation is a relation of possible or actualized causation, neither when they introduce this relation in Section 3 nor when they use it for the grounding base of spacelike and timelike distance in Section 4. Indeed, the most explicit comment they have about this is as part of a remark about the modal structure built into causets or “total causal structures":

Total causal structures are rule governed just when there are physical laws that dictate not just which events happen to be causally connected within that structure, but also which events can or cannot be connected. Thus, a total causal structure in the relevant sense is not just a pattern of actual causal connections, it is also a pattern of causal connectibility (Baron & Bihan, 2023, 11).

What they are here stating is that all nodes that are related by links in the causet are *actually* causally connected and that those nodes are, furthermore, the only nodes that could be actually causally connected. They state here, in other words, that actual causation and causal connectibility are coextensional in the total causal structures that they, on the next page, use as ground for spacelike and timelike distance.^15^

## The background radiation argument

Baron and Le Bihan’s justification for rejecting that there are timelike but not actually causally related events is their background radiation argument. This argument, to repeat, is that background radiation is so pervasive that “actual signals are systematically propagating between any timelike separated events." Background radiation denotes electromagnetic radiation that, in the geodesic optic limit, propagates on light-like geodesics. But at this level of consideration, their argument must immediately fail: two events that are timelike connected are, in general, simply not light-like connected. Just think of the light-cone in a convex neighbourhood of the spacetime point *e*: the light-cone will effectively be Minkowskian, and so events to the immediate temporal future or past of *e* (i.e. time-like related points *p* in the neighbourhood of *e*) will simply not be light-like connected to *e*. The situation might be different if the universe were not only at all times filled with radiation but also with stable, proper matter all the way down, which allows for constant scattering in all directions. But the reason why Baron and Le Bihan turn to the plenum of radiation is that there seems none of that stable matter (and even less of a continuum) everywhere in the actual universe to begin with.^16^ As a result, then, the background radiation argument does not establish that all timelike separated events are actually causally related and, therefore, it fails to establish, too, that the links in the “total causal structures" that ground spatiotemporal relations are *actual* causal relations. Indeed, no non-lightlike related events can be related by an actual causal relation actualised by electromagnetic radiation.

As a consequence, Baron and Le Bihan are left with two options: Either they denounce Smart’s requirement of actual causation or they provide an argument that actually meets this demand. There are, however, some principled difficulties with the latter option. In relativity theory, timelike separated events have an infinite number of timelike curves connecting them. Because of the discreteness of causal set theory, the number of causal chains between causet nodes corresponding to timelike separated spacetime events might be finite but there will still, in general, be more than one causal chain between such nodes in the causet. This multiplicity of curves in spacetime and, correspondingly, causal chains in the causet between two timelike separated events is the reason why an account of timelike distance needs a selection procedure which, in GR, is an optimisation on the proper times of a timelike curve between them, and, in causal set theory, is an optimisation on the number of links in these chains. Without such a selection principle, there would be no well-defined time-like distance (or an infinite number of such distances, if you prefer) between any two strictly timelike separated events.

As a consequence, the problem arises that, even if one could establish that, for every two strictly timelike separated events, the corresponding causet nodes are connected by at least one causal chain that qualifies as an actual causal connection. With there being many causal chains, the optimisation will, however, in general not select a causal chain between the two nodes that is an actual causal connection. To meet Smart’s demand for actual causation, one would either have to argue that all causal chains in the causet are instances of actual causation or limit the scope of the optimisation to only those causal chains that are actually causal.

Limiting the scope of the optimisation to chains of actual causation would have rather peculiar consequences. Two timelike separated events in two identical spacetimes could have a different timelike distance depending on which of the identical timelike curves between them were actually causal.

This does propose, though, that the demand for actual causation might be salvaged if one can argue that, for every pair of strictly timelike separated events, the optimal causal chain features actual causation. We can, however, think of no good reason why that would be so in the actual world or, even, in any relevant class of possible relativistic spacetimes. We ultimately think that this forces any causal set theory-based causal theory of spacetime to abandon Smart’s demand for actual causation.

Might, however, the background radiation argument at least answer Smart’s original objection of acausal (in the sense of not actually causally connected), but spatiotemporally (and thus of course technically causally connected) related events, such that we might be optimistic that another type of causal theory of spacetime could succeed based on this argument? To answer this objection, background radiation would have to entail that, for every spacetime event, this event is actually causally related to another event. Whether this is indeed entailed by background radiation is hard to tell because of ambiguities relating to what is being asked and ambiguities about what physics to take into consideration.

The ambiguity about what is being asked or, really, what is required of an answer arises if one wants the events to be connected by a traceable point entity. Such point entities are already problematic in general relativity, where a simple delta Dirac approach is usually not taken to work. In quantum field theory, they are outright impossible because of the absence of a well-defined position operator. The consensus in models of both theories is that there are no perfectly localised entities. If the entities of a model (if there even are such entities) cover more spacetime events, then a question is whether all events covered by the entities should be considered actually causal as a result of the causal influence exerted by those entities. If one insists on actual causation between events due to traceable point entities, then our current physics suggests that that is impossible.

The ambiguities about physics concern how to model background radiation. In the standard cosmological model, radiation is often *modelled* as a perfect fluid, and thus treated as a smooth, pervasive component of the cosmic energy density. However, from the standpoint of kinetic theory, radiation is described by a photon distribution function, corresponding to a finite photon number density. Based on the observed background radiation temperature, this number density is $\simeq 410~\mathrm {photons/cm^{3}}$. That is, there are many fewer photons than events in space as foliated by FLRW. Notably, this photon density is still many orders of magnitude below the Planck-scale densities at which a putative discreteness of spacetime (as envisioned in causal set theory or other quantum gravity approaches) would become relevant. The smoothness of the fluid model arises only as a coarse-grained approximation.

The question, however, is really how the background radiation argument applies to Baron and Le Bihan’s grounding base, i.e., to causal set theory. If one takes causal set theory to apply at the Planck scale (or similarly)—maybe in some quantum version even —, there is way too much physics in between what looks at some coarse-grained level of GR as pervasive radiation, and what the matter sector looks like at the quantum gravity scale to make any serious philosophical assessment here.

Suppose one takes (non-standardly) CST to apply at scales of GR, as some form of discretised GR perhaps. In that case, one may indeed be able to make the case that for some causets (namely, those corresponding to FLRW spacetimes), there is pervasive radiation. But this is in a way just the same point one can make with respect to certain GR spacetimes already, such as FLRW. All that would follow from this is that some (high-idealised) causets (or also already GR spacetime scenarios) could be taken to be full of actual causal relations due to background radiation; but the coarse-grained and idealised nature of these would make the implications on philosophical grounds uninteresting.

But leaving their speculations on physics aside, the background argument seems, in any case, ill-fitted as a remedy for that actual causal relatedness, as mediated via background radiation, corresponds to the sort of actual causation that Smart (see quote above) or anyone in search of a more philosophical sense of causation is after. Put simply, mediation via radiation seems for the relation between two timelike events (say, between me throwing a ball, and the ball smashing a window) for all practical purposes completely irrelevant. More concretely, Baron and Le Bihan propose to think of the causal set relations as encoding actual causal relations in Woodward’s interventionist sense (Woodward, 2005), first and foremost in the spirit of Pearl and Causality (2009) and Frisch (2014) who discuss interventionism for causal graphs:

An intervention into the variable $X_i$, according to Pearl’s account, then consists of removing the structural equation for $X_i$ from the causal model and replacing it with an equation that fixes the value of $X_i$ to some fixed *x*. That is, formally, an intervention is represented by removing the equation $x_i = f_i(p a_i, u_i)$ from the model and replacing it with some $x_i$. Pearl calls such an intervention an “atomic intervention,” which can be denoted by “do($X_i = x_i$)" or “do($x_i$)". (Frisch, 2014, 95)

Now, Baron and Le Bihan “are prepared to admit that we are talking about a different notion, one that does apply at the fundamental level and that shares certain continuities with the notion that interests Woodward and Pearl." (p. 21). For that reason, they seem to anticipate that “the kind of dependence that we use to rebuild the causal theory might not be considered as causal by everyone." (p. 21), and that one might also just think of it as a “modal theory of spacetime". However, what is interesting is that one might simply question whether these notions even remotely apply here. Interventionism (in general) rests on the idea that change in the supposed cause goes along with a change in the supposed effect. It is not clear to us that such a statement can be made with respect to background radiation. They give an example here: “think of you raising your hand right now and the burst of a supernova in galaxy Andromeda in the far distant future; there is a sense in which the event in Andromeda will be affected in a negligible way by the modifications of the field caused by you raising your hand." (Baron & Bihan, 2023, 206) It is not clear to us that physics will ever have anything to describe about this “negligible way” how one affects the other here at all; we expect however not at a story at the level of GR or that of its classical discretisation without matter fields (as the causal set theory we get presented by the authors presumably captures) to account for this; and most importantly we do not understand why this negligible way (which is suggested as something one can simply de-idealise but is simply beyond scope of all theory these days) could ever be of interest at all beyond the verbal and towards something fruitfully instrumental: after all, of course, one can stipulate radiation to be a difference maker of causal, modal or whatever character but by calling events in the causal back light cone of an event a difference maker you end up with a trivially large class of difference-makers—namely everything in the backwards lightcone, and with the need to set up a more narrow notions again. There is nothing fruitful in this conceptualisation, and thus no good motivation for upholding it.

## Reviving the identity theory

Even if the non-identity causal theory of spacetime has its problems, it might still be an improvement on identity theories: All the problems raised above would be equally detrimental to an identity theory based on causal set theory. At the same time, prima facie, the identity is worse off. As Baron and Le Bihan insist, spacelike separated events are a problem for an identity causal theory of spacetime because

Those events, in order to be causally related, would need signals propagating faster than the speed of light in a vacuum, which is physically impossible according to the general theory of relativity. [...] it follows that there are no such relations with which the relevant spacelike separations can be identified (Baron & Bihan, 2023, 5).

The problem is that spacelike separated events cannot—according to one convention by definition—be causally connectible. Because the causet approximating a spacetime must observe its causal structure through the *≺ * relations, spacelike separated events cannot be related by the *≺ * relation. Non-identity theory is a solution to this problem because, “In the case of the non-identity theory, we are not required to find causal relations between spacelike separated events that can be identified with the spatiotemporal relations between them" (Baron & Bihan, 2023, 10). This is because, “Unlike identity, grounding allows for a relation at one level to be grounded in the absence of a relation at another level" (Baron & Bihan, 2023, 10). The idea, in other words, is to *ground* spacelike relations in the absence of causal relations because one cannot identify something with absence.

Baron and Le Bihan immediately recognise, however, that this condition is too weak. As they say, abstract objects, if they exist, would then be spacelike related on the assumption that abstract objects are causally idle. To remedy this, Baron and Le Bihan propose that, for two events to be spatiotemporally related, “they must both be parts of a total causal structure" where “A total causal structure is a set of elements in which every element is connected to at least one other element by some causal connection" (Baron & Bihan, 2023, 11). With this condition, the problem about spacelike related events for identity theories is not that there is no chain of links in the causet between them. The problem is that none of those chains between spacelike separated events qualify as causal because they do not observe the *≺ * relation. According to Baron and Le Bihan, the advantage of the non-identity theory over the identity theory is, in other words, that one can ground but not identify spacelike relations in the absence of such causal chains. That is: while something can be grounded in nothing, it cannot be identified with it. For purposes of the argument, we will grant Baron and Le Bihan this assumption.

The most immediate way to revive the identity theory would therefore be to propose that any chain of links in a causet is a causal relation. The consequence of taking this view would be that two events, *x* and *y*, can be causally related even though *x ⊀ y* and *y ⊀ x*. This, of course, is not what Baron and Le Bihan mean by ‘causal relation’ since they assume that this relation only obtains between events related by the *≺ * relation in causal set theory. However, the *≺ * relation is at the outset just an abstract mathematical relation. Baron and Le Bihan argue that those and only those events related by the *≺ * relation are actually causally related in our world, but Section 5 questioned the soundness of that argument. In the actual world and most, if not all, of the possible worlds of relativity theory, then most instances of the *≺ * relation are mere relations of causal connectibility. As discussed in Section 4, Smart did not consider mere causal connectibility appropriate for a causal theory of spacetime. Thus, insisting that events related by the *≺ * relation qualify as causally related is already a departure from the original aspirations of a causal theory of spacetime. This suggests that there is at least a discussion to be had whether we might not just as well depart from the original aspirations and say that any chain of links in the causet is a causal relation.

That we should be liberal in what we consider causal relations is further supported by considering how spatiotemporal relations are technically recovered from the causet. This is not done one spatiotemporal distance at a time. What is recovered (or is supposed to be recovered) in the practice of causal set theory^17^ is instead a spacetime as a whole from a full causet. Given a spacetime, all spatiotemporal relations are given. Likewise, given a causet, all chains of links between events are given, including those that qualify as being causally connected. Assume that if a causet recovers a spacetime, then the map is one-to-one. In this case, the spacetime could arguably be considered identical to that causal set, which is precisely a *causal* structure because it is defined only in terms of *x ≺ y*, i.e., in terms of a relation that Baron and Le Bihan consider causal.

That this holistic approach takes priority is signified by the fact that, while every spatiotemporal relation in a spacetime corresponds to a specific chain of events in the causet, there is no way to get either relation if one is only given the subcauset consisting of that chain of links. A chain of links only corresponds to a spatiotemporal relation via its place in the causal set and the mapping from that causal set into a spacetime. Jaksland and Linnemann (2024) defend in more detail the priority of recovering the spacetime over recovering its individual spatiotemporal relations. If they are correct in this assessment, then the debate between identity and non-identity theory should perhaps not proceed at the level of individual spatiotemporal relations. And if the holistic perspective is adopted, the identity seems to fare just as well as the non-identity theory in the sense that all the spacetimes that can be grounded in a specific causet might, for all Baron and Le Bihan say, be considered identical to that causet instead.

One might insist that even if the spacetime as a whole is identical to the causal set, then the causal indolence problem still shows that spatiotemporal relations cannot be identical with causal relations. In that case, though, identity must be failing for intralevel reasons. By this, we mean that the failure of identity would, in that case, appear not to arise between general relativity and causal set theory. As a consequence, the need for grounding would have nothing to do with the causal theory of spacetime but rather with the internal dependence between parts and wholes in causal set theory, and/or general relativity.

An alternative way of reviving identity theory is to propose that Baron and Le Bihan are too restrictive when they insist that identity theory must identify spatiotemporal relations between two events with a causal relation between those same events. The reason that Baron and Le Bihan impose this constraint is, as explained above, that they assume that one cannot identify something with absence, whereas one can ground it in absence. However, a problem even with grounding the spacelike relations in the absence of a causal relation between those two events is that an absence can, at most, ground the fact that two events are spacelike separated. Any *difference* in spacelike distance between two pairs of events cannot be grounded in this absence. Perhaps as a consequence of this, Baron and Le Bihan ultimately ground spacelike distance in a causal chain after all. More precisely, Baron and Le Bihan propose that “The distance between any two spacelike separated points is then grounded in the minimum number of relations of causal precedence it takes to arrive at a common causal ancestor within the underlying causal set description" (Baron & Bihan, 2023, 12). With this construction, however, they seem no longer to be grounding spacelike distance in the absence of a causal chain but instead in the presence of a causal chain between one of the two events at a spacelike distance and a third event, their common ancestor. We might therefore wonder why this construction is not available to identity theory, too.

There are overall two degrees at which one can lessen the constraint that Baron and Le Bihan impose on the identity theory. Thus, we have three different constraints that we might impose on identity theory:

1. The spatiotemporal relation between two events must be identified with a (physically possible) causal relation between those two events.
2. The spatiotemporal relation between two events must be identified with a (physically possible) causal relation that involves at least one of those two events.
3. The spatiotemporal relation between two events must be identified with a (physically possible) causal relation between two events in the same spacetime, but not necessarily those that are spatiotemporally related.

Baron and Le Bihan impose constraint (1) on identity theory and the grounding version of constraint (2) on their non-identity theory.

Constraint (3) seems not to be on the table and, as it stands, it might come across as too weak. Smart, for instance, can be read as implicitly taking this view when he rules out an identity causal theory of spacetime on grounds of the conceivability of acausal events. If the spatiotemporal relations that these acausal events stand in could be identified with causal relations between completely unrelated events, then even Smart’s scenario would not be a problem. The intuition against (3) is arguably that it simply cannot be the case that a spatiotemporal relation between two events might be accounted for by a causal relation between two events in a completely different part of spacetime.

Baron and Le Bihan already implicitly address a concern like this in their application of (2) when they add that the third event in question must be a common ancestor of the two spacelike related events. Common ancestors are not completely arbitrary events. Rather, Baron and Le Bihan stress that there is an “indirect connection between the two causal set elements that goes through a common ancestor" (Baron & Bihan, 2023, 12). What is important is that there is a causal relation between both events and this third event. What Baron and Le Bihan seem to propose is that it is legitimate to account for the spacelike distance between two events in terms of one of those events and a third event, if that third event is indirectly connected with the events being spacelike related. Relating directly to Smart’s objection, what this indirect causal connection entails is that the spacelike distanced events are not acausal in Smart’s sense of being “neither causes nor effects of other events." Having a common causal ancestor entails that both events are effects of another event.

The question of grounding vs. identity does not, however, appear as part of this discussion. Baron and Le Bihan give no reason why this argument for the legitimacy of approach (2) should be exclusive to grounding. Why can a proponent of identity not likewise *identify* the spacelike distance between two events with the causal relation between one of those events and their common ancestor? Grounding might have the advantage over identity in that one can ground in absence. Baron and Le Bihan, however, are not grounding in absence. They are grounding the spacelike distance between two events in a causal relation that is not between those two events. For all Baron and Le Bihan say, the option is still on the table to instead identify the spacelike distance between two events with a causal relation that is not between those two events.

The only problem we can see is that this construction does not actually give a plausible ground for spacelike distance for the cases where the spacelike distance between two events is even well-defined. Jaksland and Linnemann (2024) have developed this point in more detail. With respect to Baron and Le Bihan’s account of spacelike distance between two events in terms of the shortest chain between one of the events and a common ancestor, they write:

The shortest causal chain to a common ancestor will correspond to the least proper time path to a common ancestor. In the GR model, that common ancestor will be at the intersection (if there is one) of the past lightcones of the two events. Consequently, the shortest time-like path to the common ancestor will be arbitrarily close to having proper time zero. While there may be no causal chain corresponding to exactly such a path, there must be one relatively close to it to satisfy the uniformity condition on the embedding. Thus, the shortest causal chain from a common ancestor to the events corresponds to a causal path with nearly vanishing proper time. Baron and Le Bihan’s ground for spacelike distance entails, in other words, that the distance between every two spacelike separated events approximately vanishes. But whatever “spacelike distance" refers to at the level of GR, such numerical degeneracy can hardly be appropriate.

**Fig. 1:**
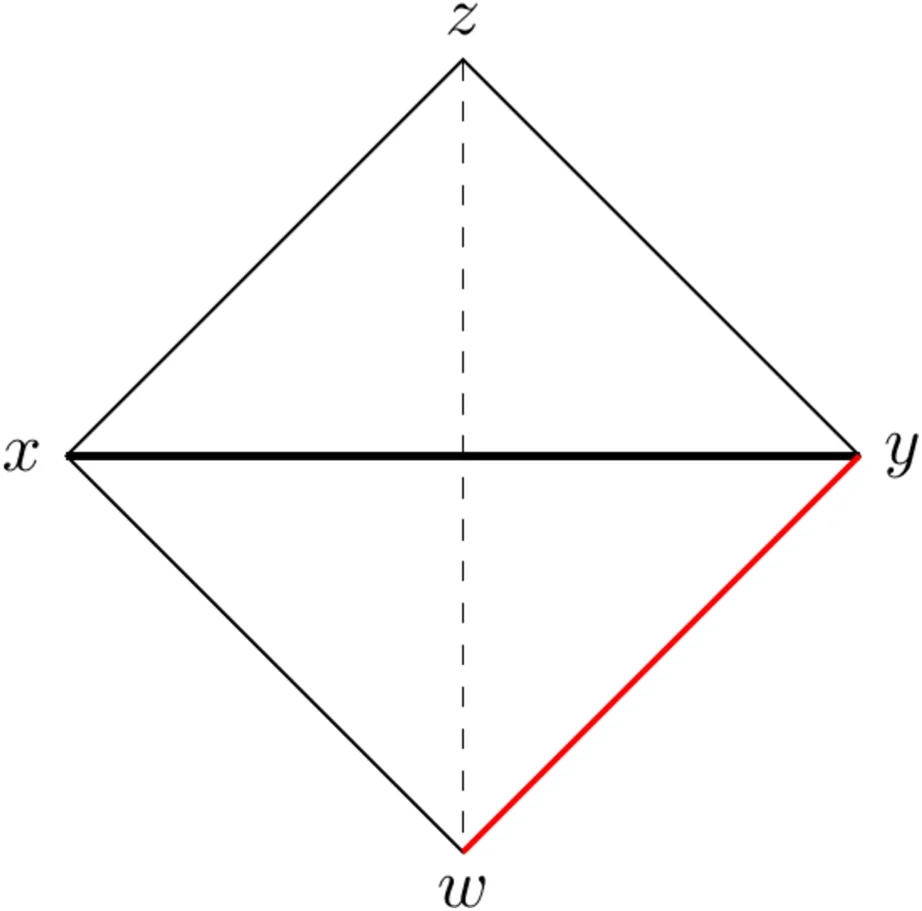
Minkowskian-style causal diamond that schematically visualises conceptions of CST-spacelike and GR-spacelike distance between events x and y: (1) Baron and Le Bihan propose a notion of CST-distance between spacelike-related events as the shortest distance possible between an ancestor w of x and y, and x or y (here chosen to be y; in the given depiction, x would equally work). This would correspond to the length of the red line between w and y in the diagram, which is always zero for GR-spacetimes, and in causets close to zero. Their conception of spacelike distance is thus pathological. (2) The standard conception of spacelike distance (not always well-defined) is instead to look for the maximal distance possible between a joint ancestor and successor of x and y. In the Minkowski-style depiction here, that is just the straight dashed line between z and w

As a charitable remedy, then, Jaksland and Linnemann propose a construction more in line with what the CST literature acknowledges as a standard counterpart to “spacelike distance" in GR (Rideout & Wallden, 2009, 5): define a ground for spacelike distance in a general relativistic model as the CST-spacelike distance, $D_s(x, y)$, with events *x* and *y* in the causet *C*, by the shortest CST-timelike distance between a common ancestor, *w ∈ C*, and a *common successor*, *z ∈ C*, i.e,

1 $$\begin{aligned} D_s (x, y) = \text {min}_{(w, z) \in \mathcal {I}} D(w, z) \end{aligned}$$

where *D*(*w*, *z*) is the (intrinsic) CST-timelike distance (a counterpart to timelike distance in GR, that just counts the number of events between *w* and *z*), $\mathcal {I} = \{ (w, z) | w \in \mathcal {J}^+(x) \cap \mathcal {J}^+(y), z \in \mathcal {J}^{-}(x) \cap \mathcal {J}^{-}(y)\}$, and $\mathcal {J}^+(x):= \{y \in C | x \prec y \}$ ($\mathcal {J}^-(x):= \{y \in C | y \prec x \}$) denote the causal future (past) of a causal set element.^18^ The notion of CST-spacelike distance and its original counterpart from GR are easily demonstrated in a ‘causal diamond’-like structure; they are both shown along with the defective proposal of Baron and Le Bihan (see figure 1).

While the corrected construction proposed by Jaksland and Linnemann bases the spacelike distance between two events in a causal relation as required, the causal relation is between two other events, i.e., this is a realisation of (3). As mentioned, (3) is implicitly ruled out by Smart as well as by Baron and Le Bihan. However, if the reasoning for accepting (2) is that there is an “indirect connection" between the two spacelike distanced events through that common ancestor, then (3) might be acceptable, too. There is likewise such an “indirect connection" between the events through their common successor (assuming, as with the common ancestor, that it exists). Indeed, the causal diamond illustrated in fig. 1 shows how the common ancestor and common successor are not completely arbitrary events but connected in a very particular way. This construction will, therefore, relate to Smart’s objection in the same way as (2), the only difference being that the events in question are now both effects of a causal common ancestor *and* causes of a common causal successor.

However, deciding whether (3) is ultimately acceptable is not our purpose here. What we have pointed out is that, to avoid being technically defective, only (3) is available to identity and non-identity theory alike. Both can at most abide by their respective versions of (3) when, respectively, grounding and identifying a spatial distance with a causal relation. We see no reason, though, that this move from (2) to (3) should compromise identity theory more than non-identity theory. The question remains whether there are principled reasons why one can ground but not identify the spacelike relation between two events in the causal relation between events that are not those same two. We can think of no such reason, and Baron and Le Bihan provide none.

## Conclusion

Baron and Le Bihan’s new non-identity causal theory of spacetime has, as shown above, certain discrepancies with the historical project of accounting for spatiotemporal relations in causal terms. This qualifies their claim that their proposal is continuous with this historical project. Be this as it may, one might reply. The comparison with the traditional aims of causal theories of spacetime has emphasised some important limitations to Baron and Le Bihan’s new non-identity theory. The non-identity theory can at most account for the spatiotemporal relations of spacetimes that can be approximated by causal sets. The fundamental relations of causal sets are, in turn, relations of possible but not necessarily actual causation. Thus, Baron and Le Bihan’s account of spatiotemporal relations in terms of relations of causal sets is, at the outset, accounting for spatiotemporal relations in terms of relations of merely possible causation. And their background radiation argument does not succeed in showing that these relations of possible causation can be thought of as, after all, actualised.

More generally, arguing that all relations of causal sets are relations of actual causation or just that this is so for all those relations that ground spatiotemporal relations looks incongruent with the physics at play. For spacetimes approximated by causal sets, Baron and Le Bihan’s non-identity theory might still account for their spatiotemporal relations in terms of the causal relations of possible causation. The comparison to the historical discussions of causal set theory qualifies, though, any advantage Baron and Le Bihan’s non-identity theory holds, in this regard, over an analogous identity theory. Essentially, any advantage results from Baron and Le Bihan’s insistence that identity theory, but not non-identity theory, must account for spatiotemporal relations between events in terms of causal relations between those same events. This requirement, however, has no counterpart in the literature that Baron and Le Bihan refer to as what originally defeated the identity theory.^19^ As we argued, if identity theory is liberated from this condition such that spatiotemporal relations between two events can be identified with a causal relation between two different events, then identity theory performs just as well as non-identity theory. Alternatively, proponents of identity theory might argue that Baron and Le Bihan have already deviated so much from the original aspirations of a causal theory of spacetime that further deviations that favour identity theory could be just as legitimate. The example we gave was to further change what qualifies as a causal relation.

In sum, any advantages gained from Baron and Le Bihan’s move to a causal set theory-based *non-identity* causal theory of spacetime seem to be advantages with respect to conditions that Baron and Le Bihan have themselves set up and not with respect to aims of the original causal theories of spacetime as conceived by Earman and Smart.

## Footnotes

1 We will not discuss whether Baron and Bihan (2023) answer the concerns of Nerlich (1982) which they, besides Smart and Earman, refer to as what defeated the identity theory, although we would claim that they do not.

2 As of now, causal theory *de facto* only provides a more or less functioning discretisation of Lorentzian spacetimes, although limited attempts at defining a (classical) dynamics exist. See Surya (2019) for a progress review.

3 See, again, Surya (2019) for a recent review; and Wüthrich (2023) (and references therein) for a philosophical presentation.

4 For details, see Surya (2019).

5 See Jaksland and Linnemann (2024) for a discussion of whether these concepts are generally meaningful in relativity theory.

6 Jaksland and Linnemann (2024) show that the optimisation procedure, if a construction like this works at all, must be maximisation and not minimisation as Baron and Le Bihan propose.

7 Furthermore, this includes the criticism by Nerlich (1982), which we will not, however, discuss ourselves in detail, either. Nerlich’s objection is fully diametral to any discussion, like theirs, of causality internal to GR-style causal structure (that is, inherited by causal set theory). Instead, Nerlich’s criticism is directed towards the assumption that relativistic causality is the relevant notion of causality. It is based on the possibility of tachyons—subluminal particles whose trajectories are spacelike paths. (As tachyons could mediate causal interaction between entities that are spacelike separated, their existence would strongly speak against the assumption that relativistic causal structure aptly captures what one means, or should mean, by causal connectibility.) Nerlich’s concern is thereby exactly that there might be ‘matter’ that does not obey that ‘causal’ structure; i.e., that a causal theory *based* on the technical causal connectibility structure of a Lorentzian manifold (or, transferred to causal set theory, causets) is not including all possible cause-effect relations (i.e., the ‘true’ causal relations) that lie beyond the scope of its narrow technical sense of ‘causal connectibility’.

8 For a critical reconstruction of Earman’s criticism, see Wüthrich and Huggett (2025). (2020

9 As Earman also notes, causal loops are not necessary for a failure of spatiotemporal coincidence—the failure of spatiotemporal coincidence only implies that there are ‘almost’ closed causal curves—i.e. that for “Every open neighbourhood (in $T_{\text {man}}$) of *x* contains an open neighbourhood which is not entered twice by any causal curve" (p. 78).

10 In fact, no non-distinguishing spacetime is compatible with an underlying causal set theory. See Wüthrich and Huggett (2025)() for discussion.

11 They talk of the microwave background radiation which, strictly speaking, only occurred upon recombination. In any case, the universe is standardly described as filled with radiation from even earlier on. We will take their point to concern background radiation more generally.

12 To be petty, ‘signal’ (in the sense of actual information-transmission) is probably not what Baron and Le Bihan should mean but rather influences that could serve as signals.

13 This raises another issue that causal set theory is not currently able to include actual signals like this.

14 Thanks to an anonymous reviewer for their suggestions on this point.

15 An alternative reading of this remark is that the total causal structure contains some links that are actual causal relations and, *in* addition, some links that are only relations of causal connectibility; however, the context of the quotation speaks against this interpretation.

16 Radiation is perhaps more pervasive than matter proper in the actual universe, but that does not mean yet that it is all-pervasive. In fact, at the level of the FLRW models (and only at that, it seems), matter—both matter proper and radiation—is modelled as a perfect fluid and so as all-pervasive. More on this below.

17 See Surya (2019) for a review on the status of this recovery.

18 The notion of CST-spacelike distance is in itself arguably not always satisfactory—see Jaksland and Linnemann (2024, 12) and Rideout and Wallden (2009, 5) for details.

19 One reason being that both Smart and Earman are discussing a causal theory of time, as evident from the respective titles of their papers, and they do, therefore, not discuss what conditions a causal theory of space might have to meet.
